# Utility of Underwater Peroral Endoscopic Myotomy with Near-Focus Magnification for Severe Submucosal Fibrosis

**DOI:** 10.1055/a-2903-0895

**Published:** 2026-07-23

**Authors:** Biswa Ranjan Patra, Nagesh Kamat, Sanil Parekh, Sharan Malipatil, Sehajad Vora, Amit Maydeo

**Affiliations:** 1 Institute of Gastrosciences 81727 Sir HN Reliance Foundation Hospital and Research CentreMumbaiMaharashtraIndia

## Abstract

**Background** Severe submucosal fibrosis (SMF) is the strongest predictor of technical failure and prolonged procedure time in conventional per-oral endoscopic myotomy (POEM). Underwater POEM (uPOEM) may offer advantages in fibrotic cases, but its effectiveness remains unexplored.

**Methods** This is a single-center, retrospective case series of consecutive patients who underwent uPOEM using near-focus magnification for achalasia with SMF between July 2024 and December 2025.

**Results** Of 153 POEM procedures, 12 patients had SMF (median age 56 [IQR 52–63] years, symptom duration 9 [IQR 7.2–10] years). Four were treatment-naive. Eight had type I (66.7%) and four (33.3%) type II achalasia. Sigmoid esophagus (S1) was present in 3 (25%), S2 in 2 (16.7%), and straight type in 7 (58.3%). The median integrated relaxation pressure (IRP) was 30 (IQR 27–33) mmHg. uPOEM was successfully completed in all 12 patients (100%) with near-focus magnification in whom uPOEM-assisted tunneling was initiated, after exclusion of one aborted pre-tunneling case. The median procedure time was 54 (IQR 52–58) min. One patient had a mucosal breach (Type I), managed with hemoclips. The median Eckardt score decreased significantly from 7 to 0 at 3 months ( *p* < 0.001).

**Conclusions** uPOEM is safe and appeared feasible in severe SMF, yielding favorable short-term outcomes. Near-focus magnification allows better identification of the submucosal layer allowing for more precise dissection.

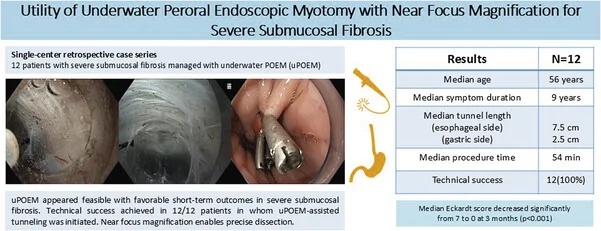

## Introduction

Per-oral endoscopic myotomy (POEM) is the treatment of choice for achalasia and other esophageal motility disorders, but severe submucosal fibrosis (SMF) from prior interventions such as Heller’s myotomy, repeated balloon dilations, previous failed POEM, or advanced disease, including sigmoid achalasia, remains a challenging scenario in clinical practice. 1 Dense fibrosis obliterates the submucosal working space; increases the risk of incomplete myotomy, mucosal injury, perforation; and significantly increases the procedure time with conventional insufflation techniques. In such cases, carbon dioxide (CO₂) insufflation may paradoxically worsen visualization by distending an already-rigid and scarred submucosal layer, creating false dissection planes and predisposing to gas-related complications such as pneumomediastinum, pneumoperitoneum, or subcutaneous emphysema.

Underwater endoscopic mucosal resection has introduced the concept of water immersion, in which the mucosa and submucosa naturally float, thus expanding the working space and improving visualization without the need for positive-pressure gas. Underwater endoscopic submucosal dissection has since been increasingly employed to facilitate dissection in fibrotic submucosal planes. Modern dual-focus endoscopes further augment this approach by offering a near-focus mode for close-range visualization, providing optical magnification with improved image clarity and enhanced color contrast. 2 Although underwater POEM (uPOEM) was proposed to reduce gas-related adverse events, 3 4 its utility in severe SMF is unexplored. Therefore, we assessed uPOEM with near-focus guided dissection for managing severe SMF.

## Methods

This is a single-center, retrospective case series with longitudinal follow up of consecutive patients who underwent POEM for achalasia cardia between July 2024 and December 2025. All procedures were performed by a single experienced endoscopist. Patients of either sex aged >18 years who had severe SMF during POEM tunneling and had at least 3 months of follow-up were included. Severe SMF was identified when the submucosal layer appeared as a white muscular structure with a complete absence of the bluish appearance. 5 We adapted the morphological criteria established for endoscopic submucosal dissection as the morphological descriptions of fibrosis remain visually and technically consistent. The primary criterion for SMF was the nonlifting sign, where the lesion fails to separate despite injection using a water jet knife and injection needle (complete failure of submucosal expansion even despite adequate volume), and absence of bluish appearance. Patients with mucosal entry site fibrosis leading to failed submucosal tunnel entry with abandoned procedure, and those with incomplete details, were excluded. Procedural variables were extracted from the electronic logs and timestamped video recordings of the procedures. To mitigate reporting bias, data collection was performed by a dedicated researcher who was not involved in the procedural decision-making. The study was approved by the Institutional Ethics Committee (approval number: IEC/OA-25/12). Patient symptoms, prior treatment, type of achalasia, Eckardt score, duration of symptoms, pre-procedural integrated relaxation pressure, total procedure duration, and procedural findings were noted.

## Description of the Technique



**Video 1** Demonstration of underwater POEM with near-focus magnification for severe submucosal fibrosis in achalasia.


**Fig. 1:**
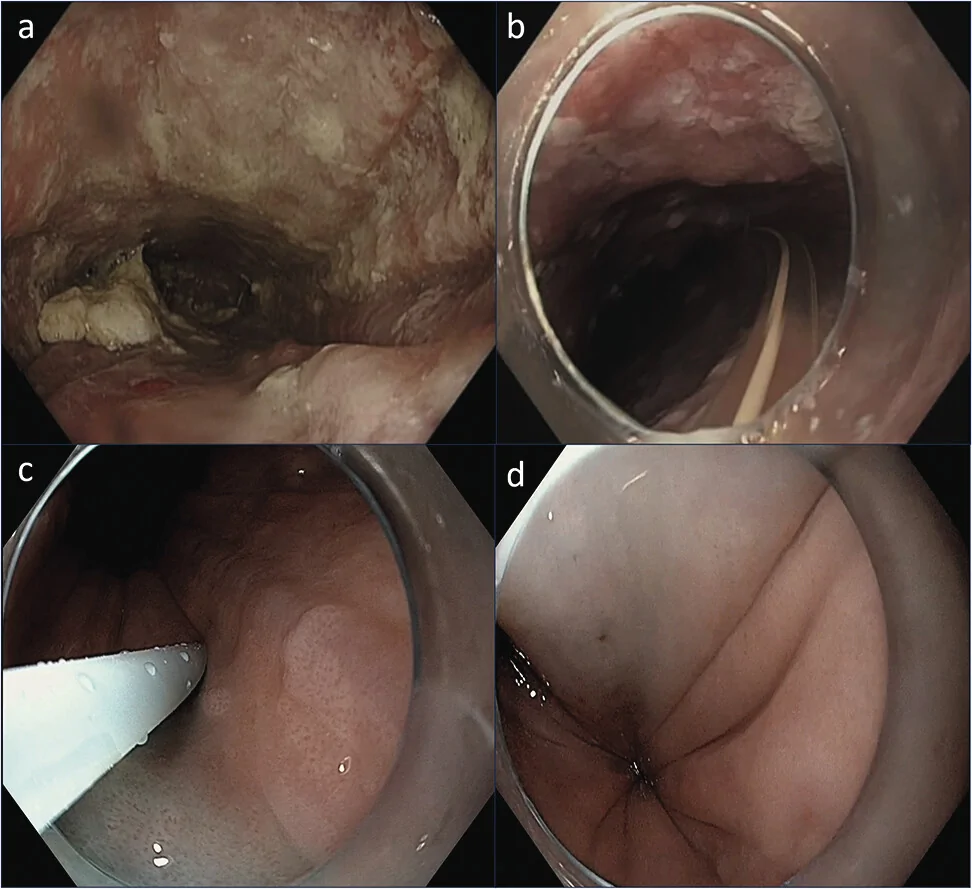
Endoscopic findings associated with difficult submucosal entry and tunneling in POEM. ( **a** ) Unhealthy esophageal mucosa with food residue. ( **b** ) Nasogastric tube placed to manage retention esophagitis. ( **c** ) Inadequate submucosal elevation or bleb formation in the posterior wall. ( **d** ) Diffuse submucosal elevation without adequate bleb formation in the anterior wall.

**Fig. 2:**
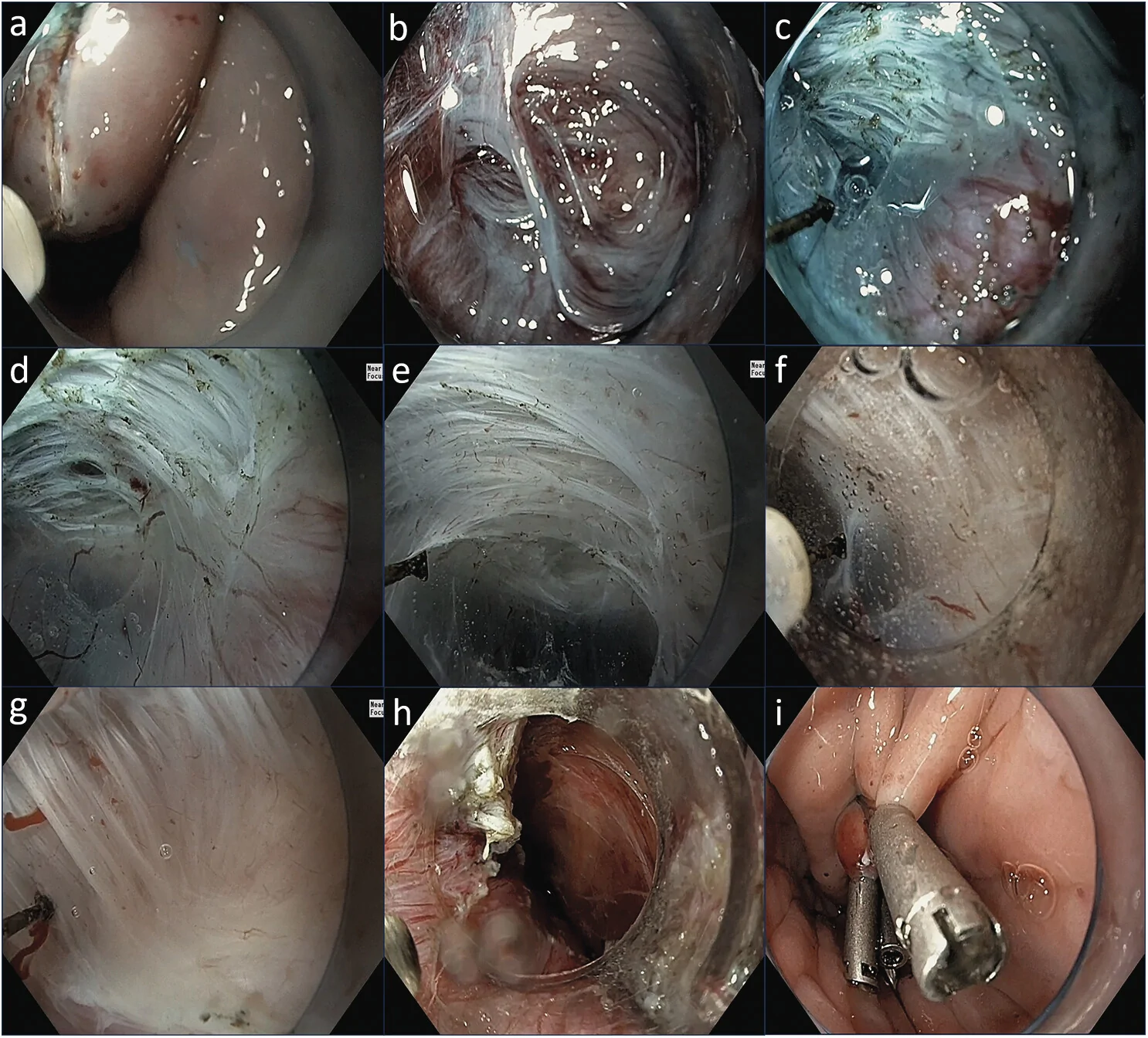
Underwater POEM in severe submucosal fibrosis. ( **a** ) Mucosal incision in the anterior wall. ( **b** ) Submucosal layer showing white muscular structure without a bluish appearance suggestive of severe submucosal fibrosis. ( **c** ) Conventional submucosal dissection under CO _2_ insufflation. ( **d** ) Switching to underwater with near-focus magnification, resulting in expansion of the submucosal plane (after failure with conventional tunneling due to severe SMF). ( **e** ) Underwater with near-focus guided submucosal dissection. ( **f** ) Underwater precoagulation of visible vessel. ( **g** ) Underwater hemostasis of a bleeding vessel. ( **h** ) Completed full thickness anterior myotomy. ( **i** ) Closure of mucosal entry site with hemoclips.

After obtaining written informed consent, all procedures were performed under general anesthesia with patients in the supine position. A standard POEM with an anterior (1–2 o’clock axis) or posterior (5–6 o’clock) approach was performed ( Fig. 1a–d ) using a gastroscope (GIFHQ190 Olympus, Japan) to the tip of which a transparent distal cap was attached (D201-11804 Olympus, USA). After injecting the submucosa with a methylene blue-saline solution and performing a posterior or anterior longitudinal mucosal incision, submucosal tunneling was done with the forced coagulation mode (effect 3) (VIO3/300D, ERBE Inc, USA). When the submucosa was severely fibrotic and the injection failed to provide a blue submucosal cushion, the approach was switched to underwater. The transition to the underwater technique was triggered by operator discretion during tunneling. The CO _2_ insufflation (UCR, Olympus, USA) was stopped, and room temperature sterile saline was irrigated into the tunnel through the waterjet channel of the endoscope. Near focus was used intermittently when there was difficulty in visualizing submucosal fibers. 6 The underwater submucosal tunnel was created and extended beyond the gastroesophageal junction (GEJ) for up to 2 cm into the stomach. Saline irrigation was stopped after completion of tunneling, and further myotomy was performed under CO _2_ insufflation. Full-thickness myotomy of the circular and longitudinal muscle fibers was performed due to severe fibrosis and difficulty in identifying muscle layers. Impedance planimetry (EndoFLIP EF-300, Medtronic, USA) guidance was used to determine the adequate length of distal myotomy, targeting a delta distensibility index (DI) of 3 mm ^2^ /mmHg. Submucosal tunneling and myotomy were performed using the Triangle-Tip J knife (KD-645L, Olympus, Japan) and the electrocautery settings (effect 3) and endoCUT I (effect 2/3 – 3). Precoagulation and any bleeding vessels were managed with coagulation forceps (Coagrasper, FD-411UR, Olympus, USA) with forced (effect 3) or soft coagulation (effect 4) ( Fig. 2a–i ). The mucosal incision was closed with hemoclips (Biorad Medisys, India) ( Video 1 ). Intra or postprocedural adverse events were documented. All patients were discharged the subsequent day in the absence of any discomfort. Technical success was defined as the successful completion of the POEM procedure. Clinical success was defined as an improvement in the Eckardt score (≤3) at 3 months.

## Statistical Analysis

This was performed using the Statistical Package for the Social Sciences (SPSS, version 26.0, Professional [IBM Corporation, USA]) for Windows. Categorical variables are reported as frequency and percentage, continuous variables as median (IQR). Pre- and post-intervention Eckardt scores were compared using the Wilcoxon signed-rank test. A *p* -value of < 0.05 was considered statistically significant.

## Results

Out of the 153 performed POEM procedures, 12 patients had severe SMF with a median age of 56(IQR 52–63) years and a median symptom duration of 9(IQR 7.2–10) years. Four patients were treatment naïve, while eight patients had multiple history of prior treatment with botulinum toxin injection, pneumatic dilation, POEM, and/or Heller’s myotomy ( Table 1 ). The median baseline Eckardt score was 7(IQR 7–8). Eight patients (66.7%) had type I achalasia, and four patients (33.3%) had type II achalasia. Based on esophageal morphology, sigmoid esophagus (S1) was seen among 3(25%) patients, and sigmoid (S2) in 2(16.7%) patients and straight type in 7(58.3%) patients. The median integrated relaxation pressure (IRP) at 4 seconds was 30(IQR 27–33) mmHg. Severe SMF was encountered for a median length of 2.5(2–3) cm. The median (IQR) delta DI was 3.6(3.3–4.1) mm ^2^ /mmHg. The median (IQR) tunnel and myotomy length on the esophageal and gastric side were 7.5(6.2–9), 5.5(5–7), and 2.5(2–2.8), 1.5(1.5–2) cm, respectively. The near-focus mode was strategically used in all patients during tunneling.

**Table 1:** Baseline characteristics of patients.

Variable	*n* = 12
Age, Median (IQR), y	56(52–63)
Gender, Male, *n* (%)	10(83.3)
Male:Female	5:1
#Previous therapy, *n* = 8 (%)	
Botox injection	6(50%)
Pneumatic dilation	3(25%)
POEM	2(16.7%)
Hellers Myotomy	1(8.3%)
Duration of symptoms, y, median (IQR)	9(7.2–10)
Chicago classification of achalasia, *n* (%)	
Type 1	8(66.7%)
Type 2	4(33.3%)
Type of achalasia, *n* (%)	
Straight type	7(58.3%)
Sigmoid type (S1)	3(25%)
Sigmoid type (S2)	2(16.7%)
Preprocedural Eckardt symptom score, median (IQR)	7(7–8)
Preprocedural Integrated relaxation pressure 4s (mmHg), median (IQR)	30(27–33)

# Some patients may have received more than one therapy.

Among the 153 patients (13 had severe SMF), three experienced significant fibrosis at the mucosal entry site. In two of these cases, changing the procedural axis (from posterior to anterior) allowed for successful entry and completion of the procedure. In one case, mucosal incision site tunnel entry could not be achieved due to SMF despite axis change, and the procedure was aborted. As mucosal entry could not be established, the uPOEM-assisted tunneling phase was never initiated; hence, this case was excluded from final analysis ( *n* = 12). The median procedure time was 54(52–58) min. Technical success with underwater and near-focus guided dissection was achieved in 12(100%) of patients. One patient who had previously undergone Heller’s myotomy had a Type I mucosal injury (mucosal breach at the GE junction), which was managed intraoperatively (closed with hemoclips). Clinical success was achieved in 12(100%) of patients at the 3-month follow-up [median Eckardt score was 0(0–1)]. There was a significant reduction in the median Eckardt score from 7 to 0 at 3 months ( *p* < 0.001).

## Discussion

Severe SMF poses a significant challenge during POEM for achalasia cardia, often leading to procedural failures or complications in conventional gas-insufflation techniques. SMF is frequently associated with prior interventions like Heller’s myotomy or repeated balloon dilations, which obliterate the submucosal space, resulting in aborted procedures due to poor visualization and increased risk of mucosal injury or perforation. 1 Our findings are hypothesis-generating observations, noting that the 100% success rate in this specific cohort suggests that uPOEM is a feasible and promising alternative for severe SMF. In our cohort, 12 out of 153 patients (7.8%) exhibited severe SMF, predominantly in those with prior treatments (66.7%), aligning with Feng et al., who proposed an endoscopic mucosal classification (EMIA) as an independent predictor of advanced fibrosis, emphasizing the multifactorial etiology, including long disease duration and sigmoid esophagus. 6 Switching from a posterior to an anterior approach was successful for two patients. Axis change is a solution to bypass localized scarring at the mucosal incision site. However, it does not help if the fibrosis continues deep into the esophageal wall further down. In contrast, uPOEM allows the endoscopist to tunnel through the entire length of the esophagus, across the GE junction, and into the gastric cardia, even when the traditional submucosal space is obliterated. The uPOEM technique addresses these issues by leveraging water immersion to naturally expand the submucosal layer without positive-pressure gas, which can distort fibrotic planes and exacerbate gas-related adverse events like pneumomediastinum.

The underwater approach enhances visualization and precision in fibrotic submucosa by creating a floating effect that separates tissue layers, as demonstrated in case reports. Switching to underwater immersion mid-procedure has been shown to resolve capnothorax and improve visibility in high-risk patients with comorbidities, such as COPD. 5 This is supported by broader reviews on underwater resection techniques, which describe how water immersion reduces halation, improves magnification for vessel precoagulation, and minimizes thermal injury risks compared to CO₂ insufflation. 4 In our study, uPOEM was adopted for severe SMF when conventional injection failed to yield a blue cushion, resulting in 100% technical success and a median procedure time of 54 min. This duration may be longer than those for standard primary POEM, but they remain within a clinically acceptable range for complex rescue cases. This efficiency stems from the buoyancy effect, which facilitates tunneling through white, scarred submucosa without creating false planes, a common pitfall in gas-based methods. 1 The buoyancy reduces effective tissue weight and countertraction forces, allowing adherent mucosal and submucosal layers to separate more readily even in a noncompliant, fibrotic environment. The incompressibility of water provides a continuous lifting effect thereby creating a natural dissection plane making the adherent mucosal layers float away from the muscle layer. The higher refractive index of water ( *n* = 1.33) than CO _2_ ( *n* = 1.0004) creates a natural magnifying effect. This enhances the visualization of micro-planes within the fibrosis, allowing for more precise knife tip placement that would be impossible under the standard magnification of a gas-filled environment. The refractive index of saline ( *n* = 1.334) is more than air ( *n* = 1.0003); refraction makes objects appear closer; the magnification effect narrows depth of field requiring more precise hand stability and distance adjustments for the endoscope to maintain sharp focus. Furthermore, several articles underscore POEM’s efficacy post-failed Heller’s, but our underwater adaptation extends this to severe fibrosis, potentially reducing the need for alternatives like pneumatic dilation.

While uPOEM may provide better visualization in fibrotic planes, the technical requirements for fluid exchange and bubble management result in significantly longer procedure times compared to standard POEM. Consequently, uPOEM for complex SMF cases aids in the clinical benefit of improved visibility while justifying the additional operative time, rather than adopting it as a universal protocol for straightforward cases. Additionally, the near-focus mode was used when close inspection was required in the tunnel. Once a clear dissection plane was established or when navigating nonfibrotic segments, we reverted to normal focus to maintain a wider field of view, better spatial orientation, to have broader awareness and navigational safety. In severe fibrosis, where standard views may be obscured, near focus helps identify subtle dissection planes, facilitating safer and more efficient tunneling. The near-focus mode provides enhanced magnification and a shorter depth of field, allowing clearer exposure of the submucosal layer during close-range dissection. 2

Several alternative strategies have been proposed to manage severe SMF during tunneling, including the double-tunnel technique, the use of advanced high-pressure waterjet equipped dissection knives, and open POEM. The double-tunnel technique involves the creation of a parallel submucosal tunnel to bypass scarred areas. While this approach is a reasonable rescue strategy, particularly in cases of fibrosis at the mucosal entry site, it has notable limitations in the setting of extensive submucosal tunnel fibrosis. Creation of a second (distal) mucosal entry may not always be feasible because of limited available esophageal length above the GEJ, particularly when the initial failed entry is already positioned close to the GEJ for a planned short myotomy, as commonly encountered in type I or sigmoid achalasia and during redo POEM. Additionally, switching between posterior and anterior approaches demands advanced expertise and is associated with prolonged procedure time. Advanced accessories such as high-pressure waterjet-equipped knives (e.g., Hybrid Knife), which are particularly useful for severe SMF, may not be universally available at all centers. Open POEM involves a direct mucosal incision followed by immediate dissection and myotomy of the muscle layer without tunneling, but has drawbacks, including increased technical complexity, a higher risk of mucosal injury or perforation due to the lack of a protective submucosal cushion, and greater demand on operator expertise. It remains poorly adopted, with limited studies and infrequent use in most centers. Hence, the uPOEM with near-focus guided dissection provides a single-tunnel salvage option.

Studies have shown that severe SMF, which prevented successful tunnel creation, was the direct cause of failure in 92.3% of aborted POEM procedures. 1 SMF was an independent predictor of mucosal injury [OR 4.530, *p* < 0.001] and severe or type II injury [OR 12.074, *p* < 0.001]. 7 Another single-center analysis demonstrated SMF as a significant predictor of mucosal injury [OR 8.33, *p* = 0.024]. 8 So, uPOEM appears to be a feasible option among available approaches for SMF. The clinical outcomes in our study, with 100% success at 3 months (Eckardt score reduction from 7 to 0), mirror the high efficacy rates in underwater adaptations for fibrosis reported by Hallit et al., where continuous irrigation improved field clarity. 4 This contrasts with conventional POEM failure rates of 10–20% in post-Heller cases, suggesting that uPOEM may facilitate adequate gastric side tunneling and myotomy in selected cases. 9 The technique’s safety is evident in the minimal complications, with one Type I mucosal injury managed endoscopically, aligning with a study that reported low adverse events in underwater resections due to the elimination of gas insufflation risks. 10

Despite promising results, uPOEM in severe fibrosis highlights several procedural nuances and potential drawbacks. Water immersion can introduce residue or bubble interference if not managed with continuous irrigation, but our use of sterile saline via a waterjet effectively mitigated this. Underwater variants require operator familiarity with immersion optics to prevent disorientation in sigmoid achalasia (25% S1, 16.7% S2 in our group). Overall, our 100% clinical success rate suggests that uPOEM is a feasible and promising alternative for severe SMF.

Limitations include retrospective design with inherent selection bias, the small sample size ( *n* = 12), and short follow-up period (3 months), which warrant prospective multicenter trials to validate our findings against those of conventional or hybrid techniques. To minimize assessment bias, clinical success was defined by objective measures such as the Eckardt Score and follow-up findings.

In conclusion, uPOEM may serve as a specialized rescue strategy for severe SMF in achalasia and warrants further evaluation in larger, well-controlled studies. Near-focus magnification may facilitate safer and more precise dissection, particularly when combined with underwater techniques. This approach builds on the established success of POEM, while addressing challenges related to fibrosis-associated procedural difficulty.

## Data Availability

All relevant data that support the findings are presented in the article.
